# Enhancing the role of innovative financing in global health

**DOI:** 10.1186/s41256-025-00463-5

**Published:** 2025-11-28

**Authors:** Zhebin Wang, Yida Wang, Yuxin Huang, Shuduo Zhou, Jian Yang, Ying Chen, Zhongfei Pei, Yunxuan Hu, Ming Xu

**Affiliations:** 1 Department of Global Health, School of Public Health, Peking University, Beijing, China; 2 Institute for Global Health and Development, Peking University, Beijing, China; 3 Harris School of Public Policy, The University of Chicago, Chicago, IL USA

**Keywords:** Innovative health financing, Global public investment, Public-private partnerships, Private sector engagement

## Abstract

Innovative financing mobilizes funds across varied channels using solidarity, Public-Private Partnerships and catalytic mechanisms to address unmet global health needs and engage investors as partners in development. As a supplementary measure to official development assistances (ODA), it offers a potential remedy for the current challenges in global health financing. Its significance has been magnified in the post-COVID era, where inadequate funding presents a significant hurdle to achieving Universal Health Coverage by 2030. Although efforts to promote innovative financing mechanisms have yielded some impactful results, existing bottlenecks hinder their widespread adoption and implementation. Collaboration among stakeholders is now imperative to foster the development and adoption of innovative financing tools in global health, necessitating the upgrading of existing methods and exploration of new avenues. We strongly advocate for engagement with the private sector, as it could revitalize investments in global health through the infusion of additional financial resources. The significance of innovation and the transformation of global health financing mechanisms also warrant attention. It is high time for all stakeholders to join forces and take actions.

## Introduction

Innovative financing has proved to be fit for purpose for mobilizing additional funds from across varied channels to meet unmet needs in global health [1]. According to the World Bank and the United Nations, innovative financing involves non-traditional applications of solidarity, Public-Private Partnerships (PPP), and catalytic mechanisms to support fundraising by tapping new and complementary sources and engaging investors beyond the financial dimension of transactions, as partners and stakeholders in the development; or deliver financial solutions to development problems [2, 3]. Inadequate financing has long been an obstacle to progress toward the goal of Universal Health Coverage by 2030. Prior to the COVID-19 pandemic, there was a huge gap in both domestic and external financing for health. The World Bank projected that the world’s 54 poorest countries—inhabited by 1.5 billion people—will face a $176 billion annual gap in their ability to pay for essential health services by 2030 [3]. The health and economic crises caused by COVID-19 have further widened this gap. Although health official development assistance (ODA) reached a record high with the disbursements of $29.1 billion in 2020 for the fight against COVID-19 and related activities, evidence has shown that the reprioritization of resources can reverse progress on health outcomes, particularly in the areas of malaria [4], tuberculosis [5], HIV [6], neglected tropical diseases, and non-communicable diseases [7, 8]. There are also concerns that this reallocation can lead to funds unavailable for new initiatives. [9]

The establishment of the Pandemic Fund marks a stride in collaborative action to reinforce health systems and mobilize resources towards the prevention, preparedness, and response (PPR) to future pandemics under the framework of International Health Regulations (IHR 2005), State Party Self-Assessment Annual Report (SPAR) and Joint External Evaluation (JEE). Promoted by the G20 High-Level Independent Panel in 2021, the Pandemic Fund closed its first Call for Proposals (CfP) in May 2023, with over $2 billion in seed capital secured from 25 sovereign and philanthropic contributors. The selected projects from the first CfP have received funding to strengthen disease surveillance and early warning, laboratory systems, and health workforce in 37 countries across six regions. In keeping with the Pandemic Fund’s mission to catalyze funding and promote coordination, the $338 million grants awarded will mobilize over $2 billion in additional resources, adding $6 for each $1 coming from the Fund [10]. The second CfP of the Pandemic Fund was opened in December 22, 2023, with an envelope of $500 million and more rounds of funding will likely be launched in 2024-2025, subject to available resources and alignment with the Fund’s Strategic Plan. However, the current fundraising of the Pandemic Fund, which is entirely grant-funded, still falls far short of its ambitious target of $10.5 billion USD per year, described as a drop in the bucket.

Against such a backdrop, innovative and effective financing mechanisms are more significant than ever to leverage additional resources from ODA and non-ODA into the health sector. It is high time to redefine the role of innovative financing mechanisms in global health. In this article, we review the existing health financing instruments from a holistic perspective and put forward recommendations for future innovative financing mechanisms in global health.

### Global health financing and a snapshot of existing innovative financing mechanisms

Governments and multilateral partners have implemented various mechanisms for global health financing, but the existing sources and modalities alone are not sufficient enough to address the financing issues. Assessed contributions (AC), which serve as the fundamental funding modality based on the economic level of sovereigns and are mandated by treaties, have provided base funding for several UN agencies and global initiatives. However, scaling assessed contributions in line with growing needs has proven more difficult, leading many organizations to seek voluntary contributions, primarily earmarked for specific purposes, to cover their expanded scope. As reliance on voluntary contributions increases, which, for example, has already accounted for over 80% of WHO’s budget, concerns have arisen regarding the erosion of collective decision-making and coherent organizational strategies through these “extrabudgetary” contributions [11]. While WHO receives financial support from assessed contributions, voluntary contributions, and the WHO Foundation—and is actively seeking replenishment to bridge funding gaps—the Global Fund to Fight AIDS, Tuberculosis and Malaria (the Global Fund), Gavi, the Vaccine Alliance (Gavi), the Coalition for Epidemic Preparedness Innovations (CEPI), and other large-scale financing mechanisms developed over the past two decades have relied on replenishment cycles to keep themselves afloat, which solicit fixed multi-year pledges, mainly from traditional donors. Despite that approach, most funding is still from ODA provided by a limited number of countries, rendering the replenishment approach insufficient to increase overall ODA significantly. Philanthropic donations, such as those from the Gates Foundation and Wellcome Trust, are a vital source of global health financing that should not be overlooked. The BMGF is now WHO’s second-largest contributor, having invested $751 million in WHO’s 2020-2021 budget, exceeding all other developed economies except Germany ($1.27 billion) [12]. However, it is crucial to recognize that resources from private foundations are typically earmarked and may exhibit a higher dependency on the stock market. Additionally, they may have financial interests that could potentially conflict with their grantees’ stated missions. [13]

**Fig. 1:**
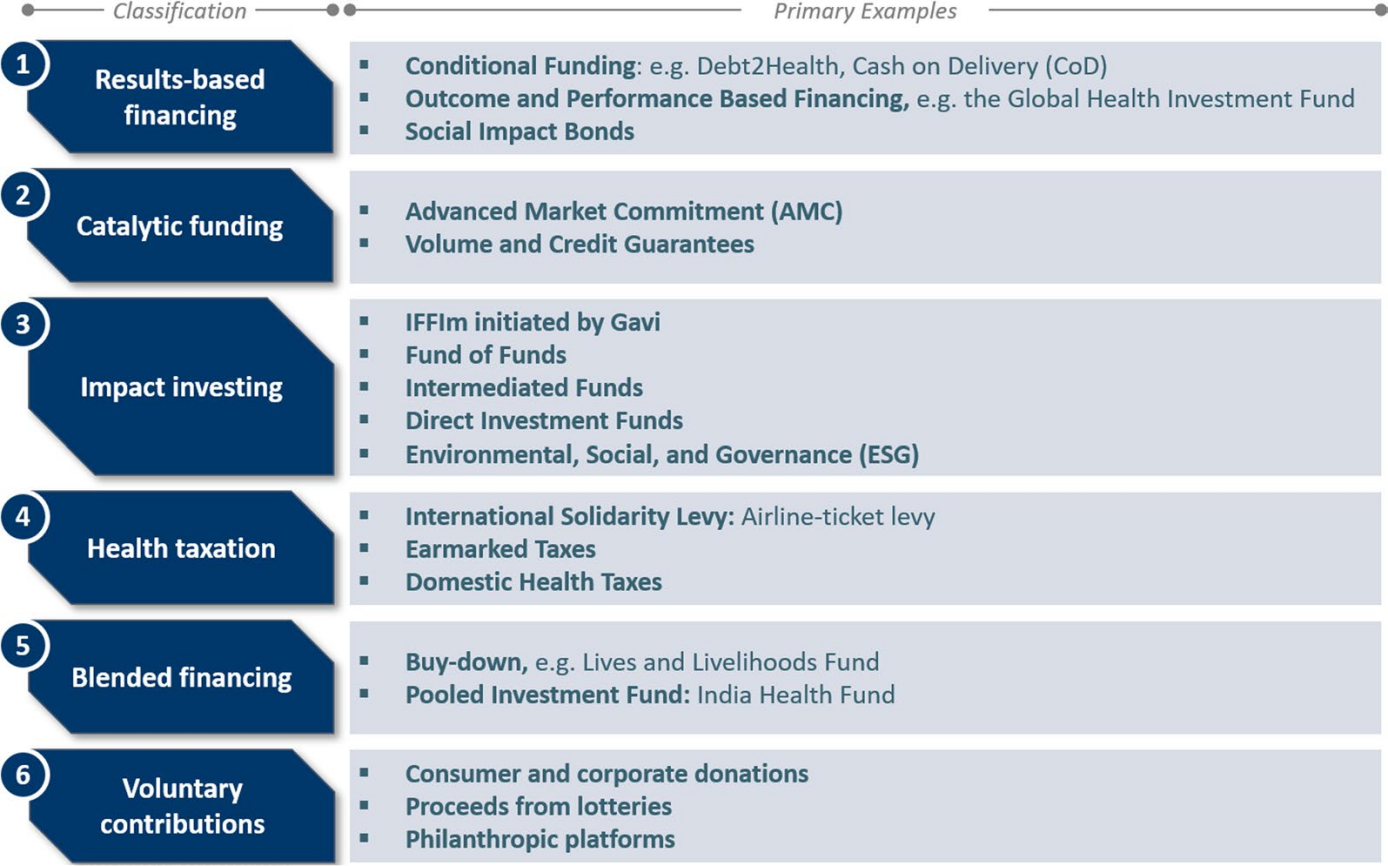
Classifications of innovative health financing mechanisms and their exemplary tools

Previous studies have conducted a comprehensive mapping of significant innovative financing mechanisms and initiatives, with a focus on health and well-being in alignment with the United Nations Sustainable Development Goal (SDG) 3 [14]. These mechanisms were developed in diverse contexts, led by various actors and aimed at addressing various health issues. Building on these previous efforts, we have further categorized these mechanisms into six key classifications: results-based financing, catalytic funding, impact investing, health taxation, blended financing and voluntary contributions, as shown in Fig. 1 [1, 15–19].

There has recently been a paradigm shift in innovative financing mechanisms from resource mobilization to other benefits, which include: a focus on results as many of the mechanisms link investment or payment to outcomes; collaboration between the public and private sectors to deliver development outcomes at scale; addressing specific market failures, for example, access to finance; more effective distribution of delivery and financial risk; and catalyzing political momentum to co-ordinate resources more effectively [20]. This change began in the early-2000s, with the creation and expansion of global instruments such as the Global Fund, Gavi, and Unitaid. These initiatives signaled a transition from a narrow focus on resource mobilization alone to a broader emphasis on outcomes, collaboration, and risk-sharing. This evolution is directly relevant to our discussion, as it highlights the need to reconfigure financing mechanisms to address today’s more complex global health challenges.

### Achievements and limitations featured by the existing innovative financing mechanisms

Innovative financing mechanisms have succeeded in tapping into new sources of funds and involving new partners for global health. Ten innovative financing instruments, including International Finance Facility for Immunisation (IFFIm), Advance Market Commitments (AMC), Debt2Health, and others, mobilized approximately US$8.9 billion between 2002 and 2015—about 2.3% of overall development assistance for health during that period [1]. One notable example within these instruments is the Unitaid solidarity levy, which is a small tax on airline tickets (typically set at a rate of €1 per economy-class ticket and €40 euros per business-class ticket) collected from participating countries to support the development and distribution of medicines, diagnostic tools and treatment for HIV/AIDS, malaria and tuberculosis. Since its first launch in 2006, more than $1 billion has been raised annually by this levy, with no adverse effect on the airline industry and tourism [17, 21].

Furthermore, innovative financing mechanisms have showcased their capacity to mobilize funds rapidly in response to public health emergencies. In December 2022, Gavi published an evaluation report concerning their initial response to COVID-19, highlighting the persuasive reasoning behind their approach, which allowed countries to utilize funds to promptly support the pandemic response efforts [15]. The rapid response of the Pandemic Emergency Financing Facility (PEF) in bridging the financing gap for the Ebola response in the Democratic Republic of the Congo (DRC) has demonstrated the flexibility of innovative financing mechanisms. On May 8, 2018, WHO was notified by DRC of two lab-confirmed cases of Ebola, which eventually ended on June 25, 2020, as the world’s second-largest Ebola outbreak on record [22]. The PEF approved a $12 million grant towards Ebola response on May 22, 2018, just two weeks after the first cases were reported, ensuring the DRC’s three-month Ebola response plan was fully funded in 2 days with joint efforts from the government of DRC and multiple international partners [23]. However, despite this early use case, the PEF was widely criticized for failing to realize its intended potential—it was slow and complicated in later outbreaks—and was ultimately discontinued in 2021 [24]. This underlines the importance of designing such mechanisms with timely disbursement and flexibility in mind.

Despite notable achievements, innovative financing mechanisms face significant challenges that hinder their scalability and long-term sustainability. Many are donor-driven, leading to a lack of domestic ownership and dependence on external funding. High administrative and transaction costs further reduce efficiency, diverting resources away from direct health system strengthening. In some cases, financing mechanisms fail to achieve sustainable impact due to design limitations, where funds are allocated for short-term objectives without integrating into broader health financing frameworks. Additionally, certain mechanisms introduce financial risks, which may discourage broader adoption. Misalignment between donor priorities and recipient country needs further limits effectiveness, as financing instruments may not be adaptable to local economic and policy conditions. Structural constraints, including governance challenges and rigid financial regulations, also impede the ability of these mechanisms to achieve long-term success. Performance-based financing (PBF) was conceptualized as an approach that can be a catalyst towards comprehensive healthcare reform. Some scholars have argued that under the current implementation model, PBF is unsustainable in many contexts due to its donor-driven nature and lack of domestic ownership in recipient countries, with substantial opportunity costs: millions of dollars have been spent on complicated management and verification mechanisms and failed to strengthen health systems in a sustainable way [25]. Take the Global Fund’s Debt2Health program as another example. The Debt2Health scheme allows a developing country to reduce its external debt by converting the repayments into lifesaving investments in health. Despite the new source of funding this mechanism can provide, it has a relatively high transaction cost and could affect the creditability of the debtor countries since swapping debt for investment may reduce the debtor government’s ability to repay its remaining debts, potentially leading to a downgrade in its credit rating [26]. The challenge with debt relief for highly indebted developing countries is that it rarely results in increased public spending, let alone higher spending for the health sector. The reasons are manifold, including countries’ varied spending priorities and the related structural adjustment conditionalities that development finance institutions put in place for receiving support [27]. Uncompensated multilateral debt swaps could also undermine the preferred creditor status of some creditors, usually multilateral development banks, and therefore multilateral debt swaps may not be desirable from the creditor perspective [28].

It is noted that the bottlenecks faced by current innovative financing mechanisms in global health have hindered their widespread adoption. Additionally, it is important to assess whether they are bringing a net increase in overall health financing or simply reallocating existing resources towards different health areas. For example, current accounting rules allow donors to treat debt swaps as substitutes rather than complements to new funding [26, 29].

### Reconfiguration of innovative financing mechanisms in global health

There is no denying the fact that innovative financing does have the considerable potential to make a difference in global health but can only be fully realized through solidarity and globally coordinated actions that respond to the changing times. Hence, we try to introduce the reconfiguration of innovative financing mechanisms in global health from those dimensions: conceptual framework, private sector engagement, funding sources, and operating and impact models. The selection of these four dimensions reflects an integrated analytical lens derived from the challenges identified in the preceding section. They correspond to different stages and functions of innovative financing: from the conceptual design of mechanisms, to the inclusion of diverse stakeholders, to the mobilization of sustainable resources, and finally to the assessment of operational effectiveness and long-term impact. Taken together, this framework provides a systemic approach intended to address persistent financing gaps and to enhance the efficiency and effectiveness of resource utilization in global health.

Firstly, in terms of conceptual frameworks, the principle of Global Public Investment (GPI) is worth adopting for current and future financing mechanisms [30]. Its core principles are universal contribution, long-term sustainable and predictable financial commitments, fair and inclusive representation in decision-making and co-design. GPI advances current approaches to incentives and governance away from a traditional donor-recipient framework and towards a universal contribution framework, while recognizing the different capacities of countries to contribute and the different approaches they may adopt. GPI requires contributions to be made in a predictable, multi-year manner. It has also been closely integrated with domestic spending, either through co-financing arrangements or by leveraging other financial resources. Nevertheless, encouraging the acceptance of the concept among country governments, experts, civil society organizations, and various stakeholders in global health is undoubtedly demanding and may involve a substantial time frame. It requires collective efforts to effectively embrace the GPI principles and demonstrate them as feasible and workable solution.

Secondly, a deeper and broader engagement with the private sector can help to mobilize more resources and address the financing gaps. USAID has placed a strong emphasis on the private sector engagement (PSE) in global health, asserting that it can make a substantial contribution to achieving grander scale, improved efficiency, increased value for money, and enhanced sustainability in the global health arena. A successful partnership necessitates strategic coordination among all stakeholders to align the profit-driven nature of the private sector with their distinct agendas [31]. Moreover, new models beyond public-private partnerships (PPP) can be explored to involve the private sector in health investment, such as through impact investment and social entrepreneurship. (RED) stands out as an exceptional model of this innovative PPP. (RED) is an impactful private sector initiative founded by Bono and Bobby Shriver in 2006 to fight HIV/AIDS [32]. Through strategic partnerships with renowned global brands, (RED) offers specially branded versions of popular products. These (RED) products are available for purchase on platforms such as Amazon, partner retail websites, and physical stores. Every dollar generated from (RED) purchases is directed to the Global Fund, supporting crucial programs that encompass prevention, testing, counselling, treatment services, sexual reproductive health education, peer mentorship, and more. To date, (RED) has channeled an impressive sum exceeding $750 million, resulting in positive transformations for over 245 million lives. By leveraging the financial resources, expertise, and technologies of the private sector, not only a more sustainable and inclusive financing model can be created, but also the healthcare delivery, product supply, and innovation at the implementation level can be improved that benefit all stakeholders.

Thirdly, with regard to diversifying funding sources, capital markets should be fully tapped into to provide significant resources, even though investors in capital markets have limited contribution and interest in global health investment for now. Indeed, it is difficult to tap into capital markets in the conventional concept, given its profit-driven nature as opposed to the well-being of the general public. Nevertheless, previous attempts, such as the social impact bonds and health impact bonds, have demonstrated that the global health and capacity market can find a niche where both can leverage advantages from each other and be mutually beneficial. Valuable insights into the effective utilization of commercial resources can be gleaned from sectors outside of healthcare. Inclusive finance, a financial mechanism that broadens access and enhances capital distribution, sets the stage for industrial and technological advancement, while also advancing the pace of green development and industrial reform. This has significantly improved public health in eastern China, the most industrialized demographic region [33]. An investment in sustainable environmental or climate initiatives is inherently linked to addressing human-induced global health issues in developing countries such as air quality, water and food safety, and antimicrobial resistance. According to the Asian Development Bank (ADB), China’s implementation of a nationwide air pollution reduction program, financed by the ADB, Bank of Beijing, Huaxia Bank and others, has contributed not only to improved air quality but also to significant positive externalities, such as the reduction of 300 deaths per million in South Korea, according to researchers at the University of Chicago [34, 35].

**Fig. 2:**
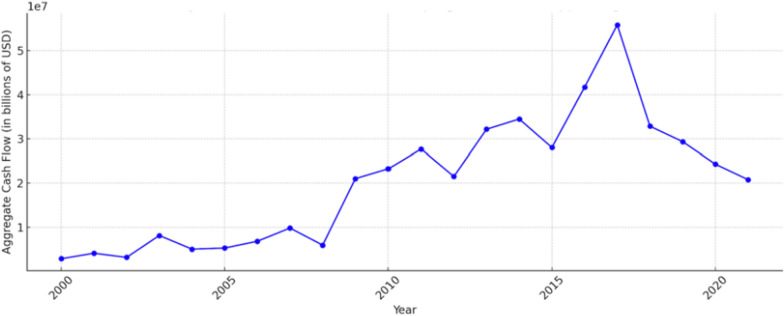
Yearly financial cash flow to developing nations in supporting ESG. Data derived from OECD and IRENA—processed by Our World in Data. This graph displays the annual total investment in clean energy R&D and renewable energy production that flows to reported countries, excluding regions and developed nations.

**Fig. 3.**
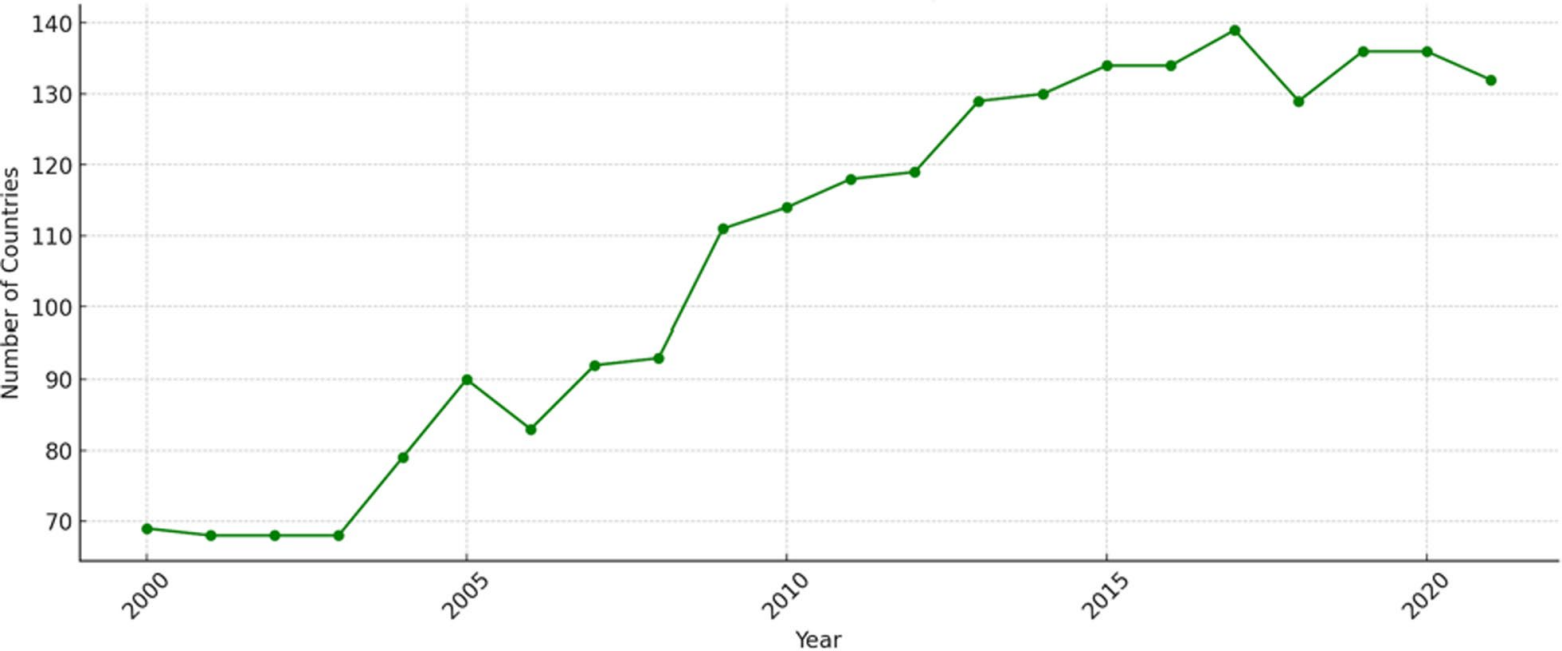
Trend of number of countries receiving positive financial flow. Data derived from OECD and IRENA—processed by Our World in Data. This graph displays the number of reported countries receiving financial cash flow, which increased significantly after 2003 and stabilized in 2015.

Fourthly, from the perspective of operating and impact models, integrating Environment, Social, and Governance (ESG) considerations into health financing could foster more responsible and sustainable investments in the health sector. The incorporation of ESG principles into pharmaceutical, healthcare, and life sciences organizations is a global trend. This trend encourages these organizations to make significant financial contributions to provide accessible medicines and therapies to underserved communities [36]. Health predominantly aligns with the social aspect of ESG, reflecting the complex interactions between corporations and communities—including employee health and safety and broader community well-being. Although public health is inherently included within the Social parameter of ESG, its explicit acknowledgment needs to be improved. Some experts have advocated expanding the ESG framework to ESHG (adding Health) in order to ensure that health impacts are not overlooked in investment decisions [37]. ESG criteria can create a more expansive model of sustainable and responsible business practices, balancing economic advancement with public health goals. ESG-oriented investment has consistently received attention in recent decades. From a governance perspective, ESG finance flows to developing countries have experienced remarkable growth since 2007, peaking around 2017. The concept of ESG brings the ‘H’ into the spotlight, emphasizing that health is not just an implicit term under ESG but a significant one that garners investor interest. As shown in Fig. 2, The recent decrease in financial flows to clean energy can be attributed to economic disruptions such as the pandemic, financial stress on utilities and energy companies, and investment patterns that disproportionately favour developed countries. However, the number of countries and regions receiving financial support has continued to trend upward, as shown in Fig. 3. On the other hand, the growth rate of ESG assets increased by approximately 34.21% from 2016 to 2018, rising from $22.8 trillion to $30.6 trillion and by about 14.38% from 2018 to 2020, reaching over $35 trillion. The Compound Annual Growth Rate (CAGR) for the entire period from 2016 to 2020 was around 11.31%. By 2025, ESG assets are projected to exceed $50 trillion globally [38].

Another perspective regarding operating and impact models is provided by Blended Concessional Finance. It has been defined by Development Finance Institutions (DFIs) as “combining concessional finance from donors or third parties alongside DFIs’ normal own-account finance and/or commercial finance from other investors, to develop private sector markets, address the Sustainable Development Goals (SDGs), and mobilize private resources.” [39] For instance, blended finance solutions of the International Finance Corporation (IFC) can be structured as debt, equity, risk-sharing, or guarantee products with different rates, tenor, security, or rank. Under select facilities, they can also be performance-based incentive structures. Solutions are tailored to address specific market barriers and failures and the requirements of donor partners. Blended Concessional Finance contributes to catalyzing market development and mobilization of private sector resources, with concessionality not greater than necessary [19]. Some of the projects supported by IFC entail high costs, inherent risks and frequently grapple with constraints related to political or regulatory uncertainties in developing countries. These constraints, in turn, limit their ability to secure funds solely from the capital markets. The IFC has been successfully implementing Blended Concessional Finance for over a decade, primarily in areas such as climate finance [18]. This approach warrants closer examination due to its adaptability to the healthcare sector. It could facilitate innovative public-private partnerships, allowing the private sector to assume an active role in resource mobilization for low- and middle-income countries. One of the fundamental principles of Blended Concessional Finance, as defined by the IFC, is to minimize the reliance on concessional funds from governments, which aligns seamlessly with the desired direction of future health financing landscape. One compelling avenue for its adoption lies in the local manufacturing of public goods for health in developing countries, such as essential medicines and diagnostics. More and more developing countries have signaled their interest in advancing local manufacturing, mainly due to the considerations of supply security, particularly in a health emergency. International organizations like WHO, the Global Fund, UNICEF, and several other global procurers have also expressed their willingness to support the production of quality assured health products to be locally made, even if they come with a higher procurement cost. Private manufacturers themselves are generally amenable to this trend, but often harbor concerns related to political instability, volatile currency exchange rates, and the associated risks in operations. In some cases, they encounter difficulties in attracting funds from the private channel. This is precisely where Blended Concessional Finance can step in as a vital facilitator, bridging the gap and providing the financial stability and confidence in investment needed to catalyze these critical manufacturing initiatives. By creating innovative financing mechanisms that attract investors and stakeholders in the capital market, the scale and impact of health financing can be potentially expanded [40].

### Policy recommendations

The evolving global health landscape can be characterized by significant investment needs, which has become more pressing than ever, in both vertically, such as focusing on a specific disease control and health issues, and horizontally, such as health system strengthening and pandemic prevention, preparedness, and response activities. Innovative solutions that can facilitate effective translation of financial outputs into health benefits and maximize their impact remain a critical challenge for the existing financing mechanism in global health. Innovations in health financing mechanisms are of critical importance against the current dynamic landscape in global health and can be approached from two perspectives. First, existing mechanisms and tools must be enriched and upgraded. It is high time to review past efforts, summarize where things fell short, and develop targeted solutions for continued improvement. Solutions can also be generated from financing mechanisms in other areas, such as the Debt-for-Nature Swap scheme in Seychelles. Second, new financing mechanisms and tools need to be created and practised in different contexts. This includes a mixture of different financing instruments into a toolkit through a multi-pronged approach. By offering a comprehensive set of options, such financing mechanisms can ensure more flexible and adaptable solutions to respond to the evolving needs and common health challenges.

We, therefore, propose four practical recommendations that require concerted efforts among all major stakeholders, including governments, international and multilateral organizations, civil society organizations, private sector actors and academic institutions. These recommendations are intentionally kept at a high strategic level rather than prescriptive operational steps, given that the primary aim of this article is to catalyze broad engagement on innovative financing and because specific implementation strategies must be tailored to each context with the evidence available.

First, build consensus. It is of utmost importance to ensure that all stakeholders reach a consensus on the value of innovative financing mechanisms in global health. At the same time, it is equally important to maximize the use of currently available resources to ensure efficiency and impact. Recognizing that relying solely on existing financing mechanisms cannot meet the growing needs posed by mounting global health challenges, all key stakeholders must adopt a more open, inclusive, and experimental attitude towards embracing innovative financing methods. A balanced approach is needed—one that not only leverages available funding more effectively but also explores new financing strategies to bridge existing gaps and enhance long-term sustainability. Toward that end, it is crucial to establish a shared understanding of the potential benefits that innovative financing mechanisms can give rise to. Additionally, approaching dialogues with transparency and evidence-based advocacy is essential. Stakeholders often hesitate to embrace new ideas due to concerns about accountability, transparency, insufficient evidence, or potential unintended consequences. By working together to achieve this consensus, an enabling environment can be created for the adoption of innovative financing mechanisms. This collaboration will empower different stakeholders to contribute collectively toward the common goal of improving health outcomes and addressing global health challenges more effectively.

Second, ensure leadership and inclusive governance. Once the consensus is reached, it is then essential to determine who should be in the driving seat to push forward innovative financing mechanisms for global health. International governmental organizations, such as WHO, and multilateral development banks, such as the World Bank, are in the right place to co-lead this effort. However, there are critical voices that question the bureaucracy and inflexibility of these institutions. New partnerships should be explored. Additionally, it is also important to build a transparent governance structure and a more inclusive and participatory decision-making process where the voices and needs of developing countries and implementation units at local can be heard and valued.

Third, generate evidence. Conducting pilots for implementation in diverse contexts can serve to test and validate the feasibility, effectiveness, and scalability of innovative financing mechanisms. These pilots enable stakeholders to identify best practices and opportunities in implementation while generating evidence that informs policymakers and other key players about the potential benefits and drawbacks of these mechanisms, thereby bolstering their confidence in their adoption. Among other things, the Pandemic Fund might introduce innovative financing mechanisms as a supplement to enhance its fundraising capacity through pilot projects. Future research can build on such pilots to assess cost-effectiveness, stakeholder acceptance, and real-world health impact, thus strengthening the evidence base for broader application.

Fourth, strengthen evaluation. We highlight the importance of evidence-based evaluation of impact and assurance of return on investment (ROI). A set of clear and standardized indicators should be involved to measure the impact of financing mechanisms on health outcomes, access to healthcare, and financial sustainability. The Global Fund has adopted several formulas and modalities, which could also be adapted to other financing mechanisms [41]. The evaluation results can be used for continuous refinement and improvement of these mechanisms, ensuring that they adapt to the evolving context and remain effective and efficient. Future applications of innovative financing mechanisms could embed such standardized indicators from the outset, enabling more consistent comparison across contexts and supporting the scaling of effective approaches.

The COVID-19 pandemic has revealed the vulnerabilities of health systems worldwide and underscored the urgent need for sustained investment in global health. Innovative financing mechanisms can help fill this gap by mobilizing untapped resources and complementing official development assistance, but their success depends on strong cross-sectoral collaboration and adaptation to local contexts. Because there is no “one-size-fits-all” model, mechanisms must be tailored to specific health systems, socio-economic conditions, and cultural realities, and they must be continuously monitored and evaluated to remain effective and sustainable. With political will, coordination, and shared commitment, innovative financing has the potential to transform health financing, advance universal health coverage, and accelerate progress toward the health-related Sustainable Development Goals, ensuring that all people everywhere have access to quality healthcare and healthy lives.

## Data Availability

Data in this study are available in a public, open access repository

## Abbreviations

AC: Assessed contributions
AMC: Advance market commitments
CEPI: The coalition for epidemic preparedness innovations
CfP: Call for proposals
DFI: Development finance institution
DRC: Democratic Republic of the Congo
ESG: Environment, social, and governance
Gavi: Gavi, the vaccine alliance
GPI: Global public investment
IFC: International finance corporation
IFFIm: International finance facility for immunisation
IHR: International health regulations
JEE: Joint external evaluation
ODA: Official development assistance
PBF: Performance-based Financing
PEF: Pandemic emergency financing facility
PPP: Public-private partnerships
PPR: Prevention, preparedness, and response
SDG: Sustainable development goal
SPAR: State party self-assessment annual report
